# Development and Validation of a Radiomics Nomogram for Predicting Clinically Significant Prostate Cancer in PI-RADS 3 Lesions

**DOI:** 10.3389/fonc.2021.825429

**Published:** 2022-01-26

**Authors:** Tianping Li, Linna Sun, Qinghe Li, Xunrong Luo, Mingfang Luo, Haizhu Xie, Peiyuan Wang

**Affiliations:** ^1^ Department of Radiology, Yantai Affiliated Hospital of Binzhou Medical University, Yantai, China; ^2^ School of Medical Imaging, Binzhou Medical University, Yantai, China; ^3^ Department of Radiology, Yantai Yuhuangding Hospital, Qingdao University, Yantai, China

**Keywords:** radiomics, clinically significant prostate cancer, PI-RADS, machine learning, prostate-specific antigen

## Abstract

**Purpose:**

To develop and validate a radiomics nomogram for the prediction of clinically significant prostate cancer (CsPCa) in Prostate Imaging-Reporting and Data System (PI-RADS) category 3 lesions.

**Methods:**

We retrospectively enrolled 306 patients within PI-RADS 3 lesion from January 2015 to July 2020 in institution 1; the enrolled patients were randomly divided into the training group (n = 199) and test group (n = 107). Radiomics features were extracted from T2-weighted imaging (T2WI), apparent diffusion coefficient (ADC) imaging, and dynamic contrast-enhanced (DCE) imaging. Synthetic minority oversampling technique (SMOTE) was used to address the class imbalance. The ANOVA and least absolute shrinkage and selection operator (LASSO) regression model were used for feature selection and radiomics signature building. Then, a radiomics score (Rad-score) was acquired. Combined with serum prostate-specific antigen density (PSAD) level, a multivariate logistic regression analysis was used to construct a radiomics nomogram. Receiver operating characteristic (ROC) curve analysis was used to evaluate radiomics signature and nomogram. The radiomics nomogram calibration and clinical usefulness were estimated through calibration curve and decision curve analysis (DCA). External validation was assessed, and the independent validation cohort contained 65 patients within PI-RADS 3 lesion from January 2020 to July 2021 in institution 2.

**Results:**

A total of 75 (24.5%) and 16 (24.6%) patients had CsPCa in institution 1 and 2, respectively. The radiomics signature with SMOTE augmentation method had a higher area under the ROC curve (AUC) [0.840 (95% CI, 0.776–0.904)] than that without SMOTE method [0.730 (95% CI, 0.624–0.836), *p* = 0.08] in the test group and significantly increased in the external validation group [0.834 (95% CI, 0.709–0.959) vs. 0.718 (95% CI, 0.562–0.874), *p* = 0.017]. The radiomics nomogram showed good discrimination and calibration, with an AUC of 0.939 (95% CI, 0.913–0.965), 0.884 (95% CI, 0.831–0.937), and 0.907 (95% CI, 0.814–1) in the training, test, and external validation groups, respectively. The DCA demonstrated the clinical usefulness of radiomics nomogram.

**Conclusion:**

The radiomics nomogram that incorporates the MRI-based radiomics signature and PSAD can be conveniently used to individually predict CsPCa in patients within PI-RADS 3 lesion.

## Introduction

Prostate cancer (PCa) is the most common malignancy cancer in newly diagnosed cancers and cause of the second cancer mortality in men (1). Multiparametric MRI (mpMRI) has become the preferred non-invasive method for the detection and assessment of PCa (2). Prostate Imaging Reporting and Data System has updated to version 2.1 (PI-RADS v2.1) in 2019 (3). The PI-RADS represents a standardized method for prostate mpMRI acquisition, interpretation, and reporting. It utilizes a 5-point scale to represent the likelihood of clinically significant PCa (CsPCa) based on the mpMRI findings on axial T2-weighted imaging (T2WI), diffusion-weighted imaging (DWI), and dynamic contrast-enhanced (DCE) maps.

The PI-RADS category 3 is the most ambiguous group in PI-RADS v2.1, which represents an equivocal suspicion of CsPCa. For PI-RADS 3 lesions, whether or not a prostate biopsy is recommended has been a matter of discussion, which depends on factors other than mpMRI alone (4), while the European Association of Urology (EAU) guidelines 2021 recommended that biopsy should be performed when MRI is positive (PI-RADS ≥ 3). Histologic biopsy examination can provide an accurate diagnosis of CsPCa, while the detection rate of CsPCa in biopsied PI-RADS 3 lesions has shown significantly high variability (range from 10.1% to 46.5%) (5, 6). It is difficult to qualitatively characterize PI-RADS 3 lesions, and how to improve CsPCa detection rate while avoiding unnecessary biopsies has always been a clinical problem that needed to be solved. Meanwhile, one of the main limitations of PI-RADS is the high inter-reader variability impacting cancer detection (7); for this reason, a radiomics model is especially useful in PI-RADS 3 lesions.

Radiomics offers a non-invasive and low-cost automated technique for the analysis of tumor properties based on MR images. It could capture large amounts of quantitative features from medical images, which could reflect underlying pathophysiology, especially tumor heterogeneity (8, 9). Radiomics could provide some guidance for clinical therapeutic decision-making by analyzing these quantitative features, resulting in improved personalization and precision medicine (10, 11). Radiomics has made great progress in the discrimination of colorectal tumors, thoracic imaging diagnosis, and some other diseases (12–14). Some radiomics studies have differentiated malignant from benign lesions and assessed the aggressiveness, survival, and treatment response in prostate lesions (15–17). However, there is limited research (18–21) applying radiomics analysis to detect CsPCa in equivocal PI-RADS 3 lesions and no validation data to verify their findings.

The purpose of this study was to develop and validate a radiomics nomogram that combined radiomics signature with clinical risk factors for individual prediction of CsPCa in PI-RADS 3 lesions.

## Materials and Methods

### Patients

**Figure 1:**
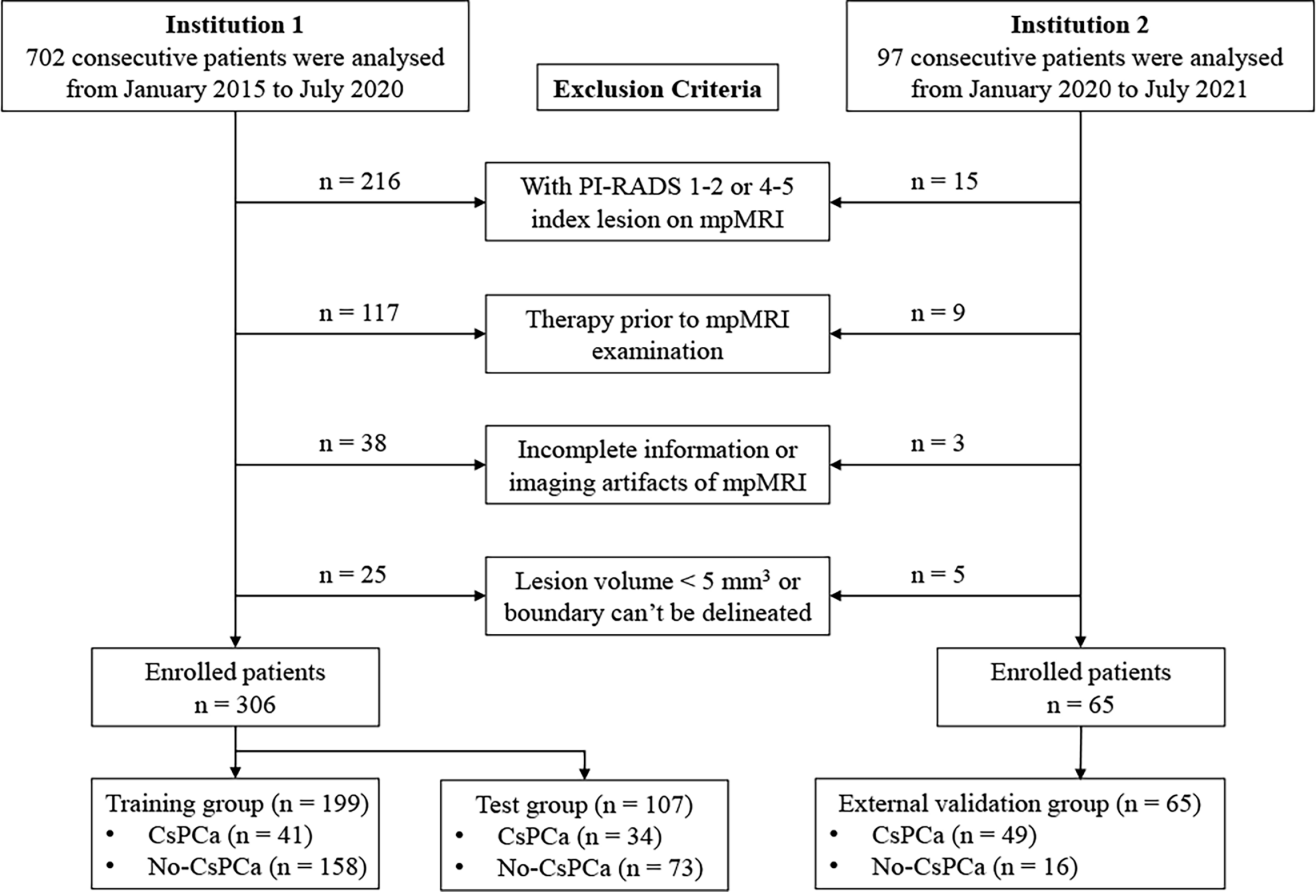
The flowchart of patient selection.

This retrospective analysis was approved by the Institutional Review Board of our hospital; patient informed consent requirement was waived. In the primary cohort, 306 patients were consecutively enrolled from January 2015 to July 2020 in institution 1. The inclusion criteria were as follows: 1) patients who underwent prostatectomy or prostate biopsy and pathological results were acquired; 2) prostate 3.0-T mpMRI examination was performed before prostatectomy or biopsy within 4 weeks; 3) serum total prostate-specific antigen (tPSA) and free PSA (fPSA) levels were measured within 4 weeks before MRI examination. The exclusion criteria were as follows: 1) with PI-RADS 1–2 or 4–5 index lesion on mpMRI; 2) prior therapy history for PCa patients including chemotherapy, radiotherapy, or prostatectomy; 3) with incomplete mpMRI information or severe imaging artifacts; and 4) lesion volume <5 mm^3^ or lesion boundary could not be delineated. The enrolled patients of the primary cohort were randomly separated into the training group (n = 199) and test group (n = 107) at a ratio of 7:3. An external independent validation group of 65 patients was enrolled in institution 2 from January 2020 to July 2021, using the same criteria as those for the primary cohort. All patients’ images were re-read by two experienced radiologists (readers 1 and 2 with 5 and 10 years of experience in MRI prostate diagnosis, respectively) following the PI-RADS v2.1 guidelines. The flowchart of patient selection is shown in **Figure 1**. Baseline clinical risk factors were derived from medical records, including age, serum tPSA level, fPSA level, PSA density (PSAD) level, and pathological results.

### MRI Examination

All patients were scanned using a 3.0-T MRI scanner (institution 1: GE Discovery MR750W, General Healthcare; institution 2: Siemens MAGNETOM Spectra, Germany) with a dedicated 8-channel pelvic phased-array coil imaging and without the use of an endorectal coil. Scan sequences included axial T2WI, DWI, and DCE images, which were compliant with the European Society of Urogenital Radiology guidelines (22). ADC maps were calculated based on DWI with b-values of 1,000 s/mm^2^ on a designated workstation. Full MRI acquisition parameters are given in **Supplementary Table S1**.

### Pathological Evaluation

All patients underwent prostate biopsy or prostatectomy. A 12-core systematic transrectal ultrasound-guided prostate biopsy was performed in our study. At least two cores were obtained from each target. The histopathological information, such as pathological type (e.g., benign prostatic hyperplasia, prostatitis, and PCa) and Gleason score (GS) were recorded. An index lesion was diagnosed as CsPCa if the pathological report GS ≥ 7 (3 + 4).

### Lesion Segmentation and Feature Extraction

Readers 1 and 2 using a free and open-source software package (3D Slicer v.4.10.0; http://slicer.org/) manually segmented PI-RADS 3 lesions. The three-dimensional region of interest (ROI) was delineated layer by layer along the lesion boundaries on the axial T2WI, ADC, and DCE images. The first enhancement phase of DCE images was chosen for segmentation. ROI was designed to not contain necrosis, cystic tissue, calcification, urethra, and seminal vesicle. An extension of PyRadiomics within 3D Slicer was performed for feature extraction (23). The following standard classes of features were extracted: shape-based, first-order statistical, gray-level dependence matrix, gray-level co-occurrence matrix, gray-level run-length matrix, gray-level size zone matrix, neighborhood gray-tone difference matrix, and wavelet transformed features.

### Intra- and Inter-Observer Agreement

The intra- and inter-observer reproducibility of radiomics feature extraction was assessed by the inter-class correlation coefficient (ICC). Initially, 30 patients’ imaging data were randomly selected for ROI segmentation and feature extraction by reader 1 and reader 2. Reader 1 then repeated the same procedure 2 weeks later. The ICCs were calculated between the features extracted from ROI by reader 1 and reader 2 and twice by reader 1. The radiomics features with poor reproducibility (ICC ≤ 0.75) were discarded before statistical assessment.

### Sample Augmentation

The model was trained and tested in the primary cohort, while the ratio of CsPCa (n = 75) and no-CsPCa patients (n = 231) was imbalanced, which would impact the performance of the model. A sample augmentation method of synthetic minority oversampling technique (SMOTE) (15, 24, 25) from the joint weighting of multiparametric features was used to address this problem in this study. The k-nearest neighbor algorithm was used in SMOTE to oversample the minority sample, until an equal number of cases in each class. The SMOTE method was not used in the external independent validation group.

### Feature Selection and Radiomics Signature Building

All radiomics features were separately normalized using the Z-scores standardization to get rid of the unit limits of each feature (26). Univariate analysis (one-way ANOVA or Mann–Whitney U test) was used to select the features that significantly associate with CsPCa. Then, the least absolute shrinkage and selection operator (LASSO) combined with 10-fold cross-validations was conducted to choose the most useful predictive features. Radiomics signature was constructed using a linear combination of selected features that were weighted by their respective LASSO coefficients, and then a radiomics score (Rad-score) was calculated for each patient. The potential association of the radiomics signature with CsPCa was first assessed in the training group and then validated in the test and external validation groups using a Mann–Whitney U test.

### Radiomics Nomogram Construction

Multivariable logistic regression analysis was applied to select independent predictors of CsPCa from Rad-score, age, tPSA, fPSA, and PSAD level, using a backward stepwise regression method based on minimal Akaike’s information criterion. Then, a radiomics nomogram was constructed based on the multivariate logistic regression model.

### Radiomics Nomogram Evaluation

Independent validation of the radiomics nomogram was performed with the internal and external validation data. The calibration curve and the Hosmer–Lemeshow test were performed to assess the calibration of the radiomics nomogram. Decision curve analysis (DCA) was performed to estimate the clinical utility of the radiomics nomogram by quantifying the net benefits at different threshold probabilities.

### Statistical Analysis

Statistical analysis was conducted with SPSS (v26.0) and R software (v4.1.1). Demographic data were compared using the independent t-test or Kruskal–Wallis H test for continuous variables, and the chi-squared test for categorical variables. Quantitative data were presented as mean ± SD or median (interquartile range) if normally or non-normally distributed. The performance of the classifier was evaluated using receiver operating characteristic (ROC) curve analysis, accuracy, sensitivity, and specificity. Delong’s test was used to compare areas under the ROC curve (AUCs). *p* < 0.05 was considered statistically significant.

## Results

### Clinical Characteristics

Patient characteristics in the two institutions are given in **Table 1**. There was no significant difference in the ratio of CsPCa between the two institutions (*p* = 0.989). The proportions of CsPCa in the two institutions were 24.5% and 24.6%, respectively. In institution 1, 70% of patients were divided into the training group (n = 199), and the rest were divided into the test group (n = 107). The patient characteristics and distribution of the training and test groups are listed in **Supplementary Table S2**.

**Table 1:** Characteristics of patients in the primary and external validation cohorts.

	Institution 1 (n = 306)	Institution 2 (n = 65)	*p*
Age (years)	70.16 ± 7.91	71.37 ± 7.27	0.257
tPSA (ng/ml)	13.53 (7.43–26.40)	17.23 (10.17–27.97)	0.072
fPSA (ng/ml)	1.63 (1.01–2.88)	2.17 (1.26–4.40)	0.013
PSAD (ng/ml/cm^3^)	0.22 (0.10–0.58)	0.23 (0.14–0.52)	0.459
Gleason score (GS):			0.012
Benign	190	47	
GS ≤ 6	41	2	
GS = 7	48	5	
GS = 8	21	10	
GS = 9	6	1	

tPSA, total prostate-specific antigen; fPSA, free prostate-specific antigen; PSAD, prostate-specific antigen density.

### Feature Selection and Radiomics Signature Building

**Figure 2:**
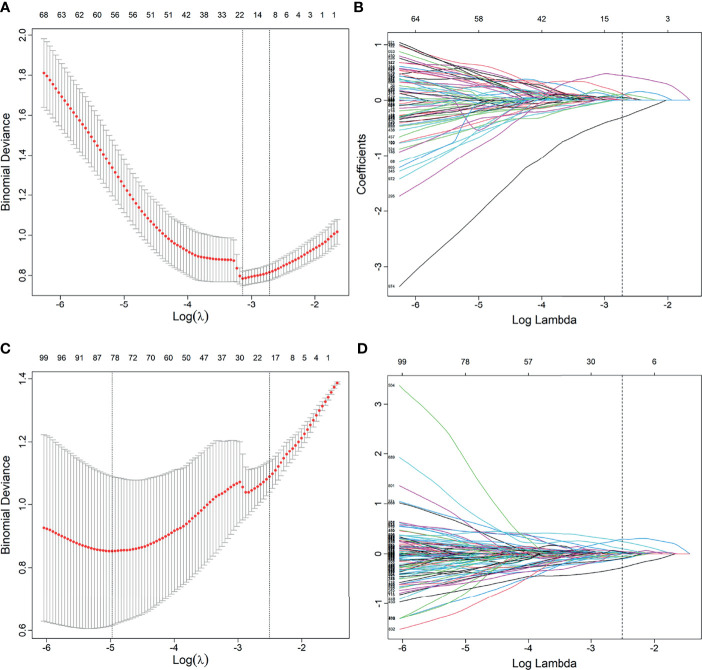
Feature selection using the least absolute shrinkage and selection operator (LASSO) regression model. **(A, B)** Feature selection in LASSO without synthetic minority oversampling technique (SMOTE) method. **(C, D)** Feature selection in LASSO with SMOTE method. **(A, C)** Tuning parameters (λ) in the LASSO model used 10-fold cross-validation *via* minimum criteria. The partial likelihood deviance was plotted versus log (λ). Dotted vertical lines were drawn at the optimal values by using the minimum criteria and the 1 standard error of the minimum criteria (the 1 − SE criteria). **(B, D)** Feature coefficients corresponding to different λ values in the LASSO model. Vertical line (optimal λ) was drawn at the value selected using 10-fold cross-validation.

**Figure 3:**
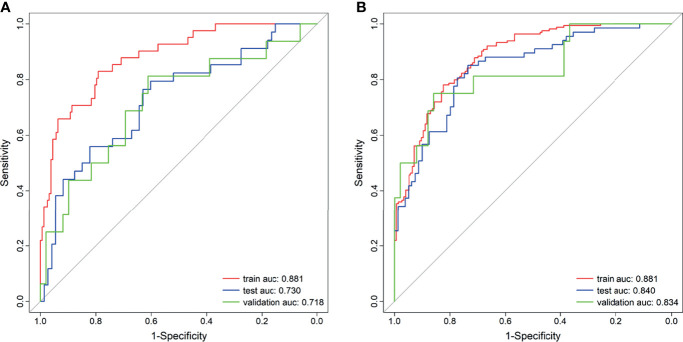
Receiver operating characteristic (ROC) curves of the radiomics signature. **(A)** Without synthetic minority oversampling technique (SMOTE) method. **(B)** With SMOTE method.

A total of 2,553 radiomics features was extracted in each patient; 2,350 features were identified as high-reproducibility (ICC > 0.75) radiomics features. Without sample augmentation, 10 features (3 from T2WI, 5 from ADC, and 2 from DCE) were finally selected from the univariate analysis and LASSO analysis (**Figures 2A, B**). The selected features are shown in **Supplementary Figure S1A**. The Rad-score was acquired and showed a significant difference between CsPCa and non-CsPCa in the training group (*p* < 0.001), which was then confirmed in the test group (*p* < 0.001) and external validation group (*p* = 0.008). The radiomics signatures yielded an AUC of 0.881 (95% CI, 0.824–0.938) in the training group, 0.730 (95% CI, 0.624–0.836) in the test group, and 0.718 (95% CI, 0.562–0.874) in the external validation group, as shown in **Figure 3A** and **Table 2**.

**Table 2:** Evaluation of radiomics signature without and with SMOTE.

	Without SMOTE				With SMOTE				*p*
	AUC	SEN	SPE	ACC	AUC	SEN	SPE	ACC	
Training group	0.881 (0.824–0.938)	0.829	0.791	0.799	0.881 (0.844–0.917)	0.780	0.822	0.801	0.982
Test group	0.730 (0.624–0.836)	0.794	0.603	0.664	0.840 (0.776–0.904)	0.851	0.734	0.788	0.083
External validation group	0.718 (0.562–0.874)	0.813	0.612	0.662	0.834 (0.709–0.959)	0.750	0.857	0.831	0.017

SMOTE, synthetic minority oversampling technique; AUC, area under the curve; SEN, sensitivity; SPE, specificity; ACC, accuracy.

When using the SMOTE augmentation method, 19 features (3 from T2WI, 11 from ADC, and 5 from DCE) were lastly selected (**Figures 2C, D**). The selected features are shown in **Supplementary Figure S1B**. The Rad-score also showed a significant difference between CsPCa and non-CsPCa (*p* < 0.001 for all the training, test, and external validation groups). Compared with that of the method without SMOTE, the performance of radiomics signature was improved with an AUC of 0.840 (95% CI, 0.776–0.904, *p* = 0.083) in the test group and significantly improved in the external validation group with an AUC of 0.834 (95% CI, 0.709–0.959, *p* = 0.016), as shown in **Figure 3B** and **Table 2**.

### Radiomics Nomogram Construction

**Figure 4:**
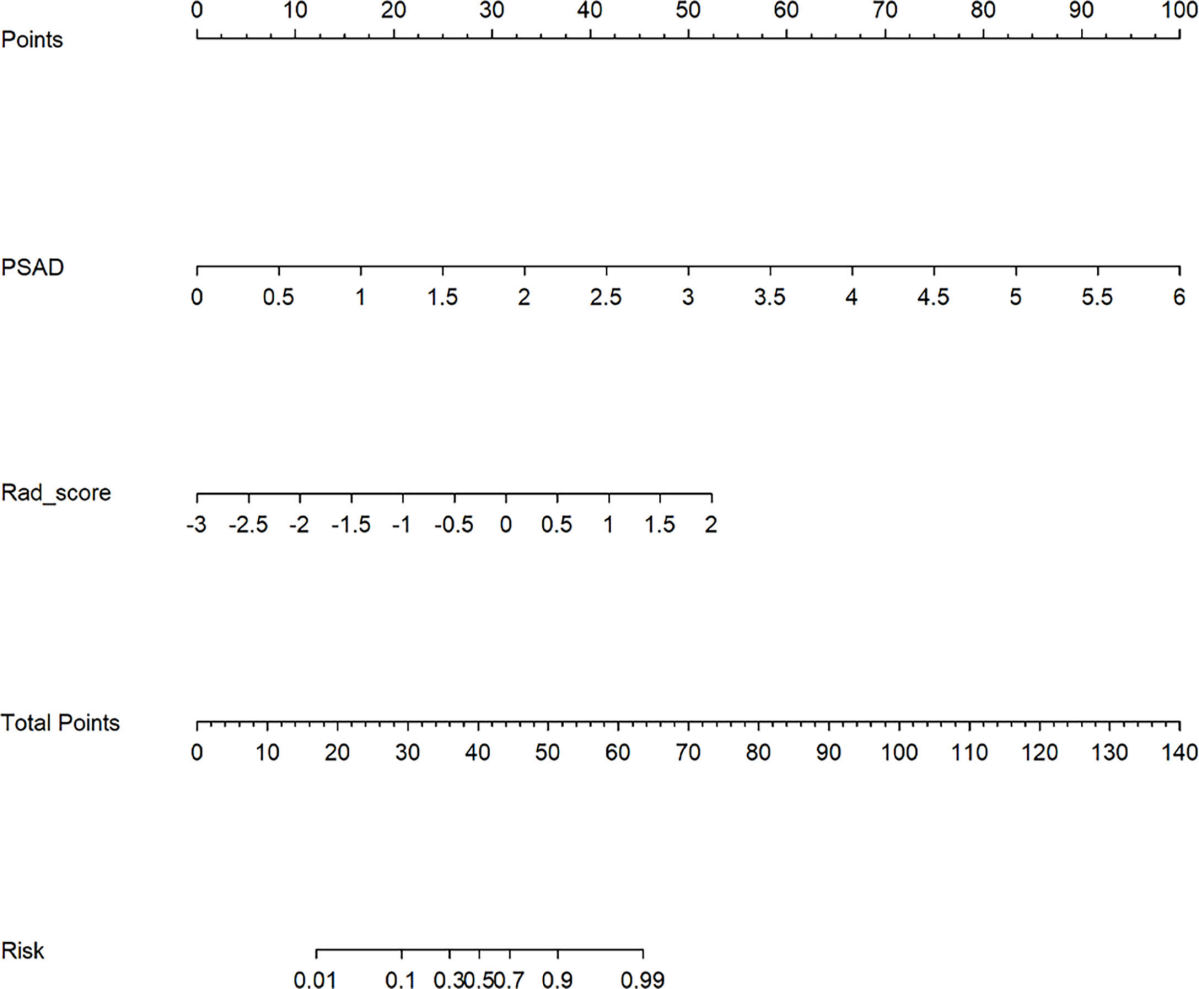
Developed radiomics nomogram for predicting clinically significant prostate cancer.

The multivariable logistic analysis of Rad-score and clinical characteristics revealed that Rad-score and PSAD level were clinical independent predictors. Then a radiomics nomogram incorporating these two predictors was developed as shown in **Figure 4**.

### Radiomics Nomogram Evaluation

**Figure 5:**
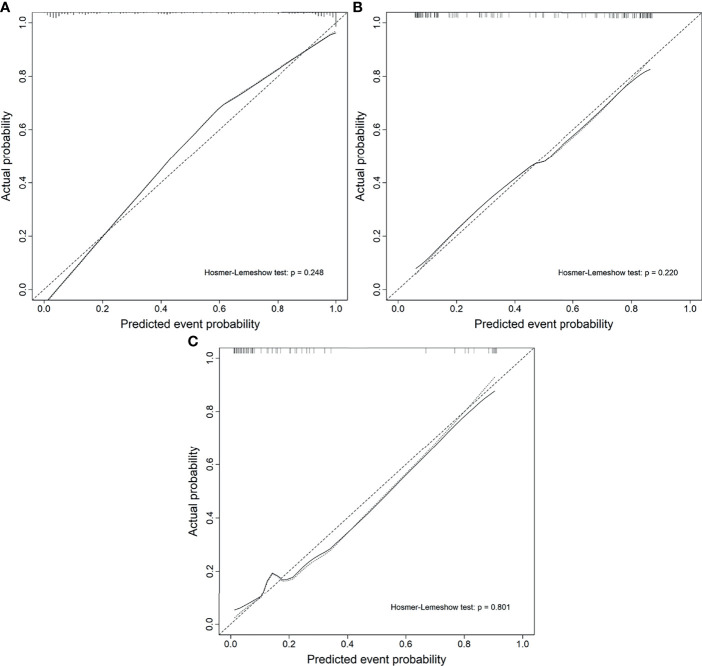
Calibration curves of the radiomics nomogram. **(A)** The training group. **(B)** The test group. **(C)** The external validation group.

**Figure 6:**
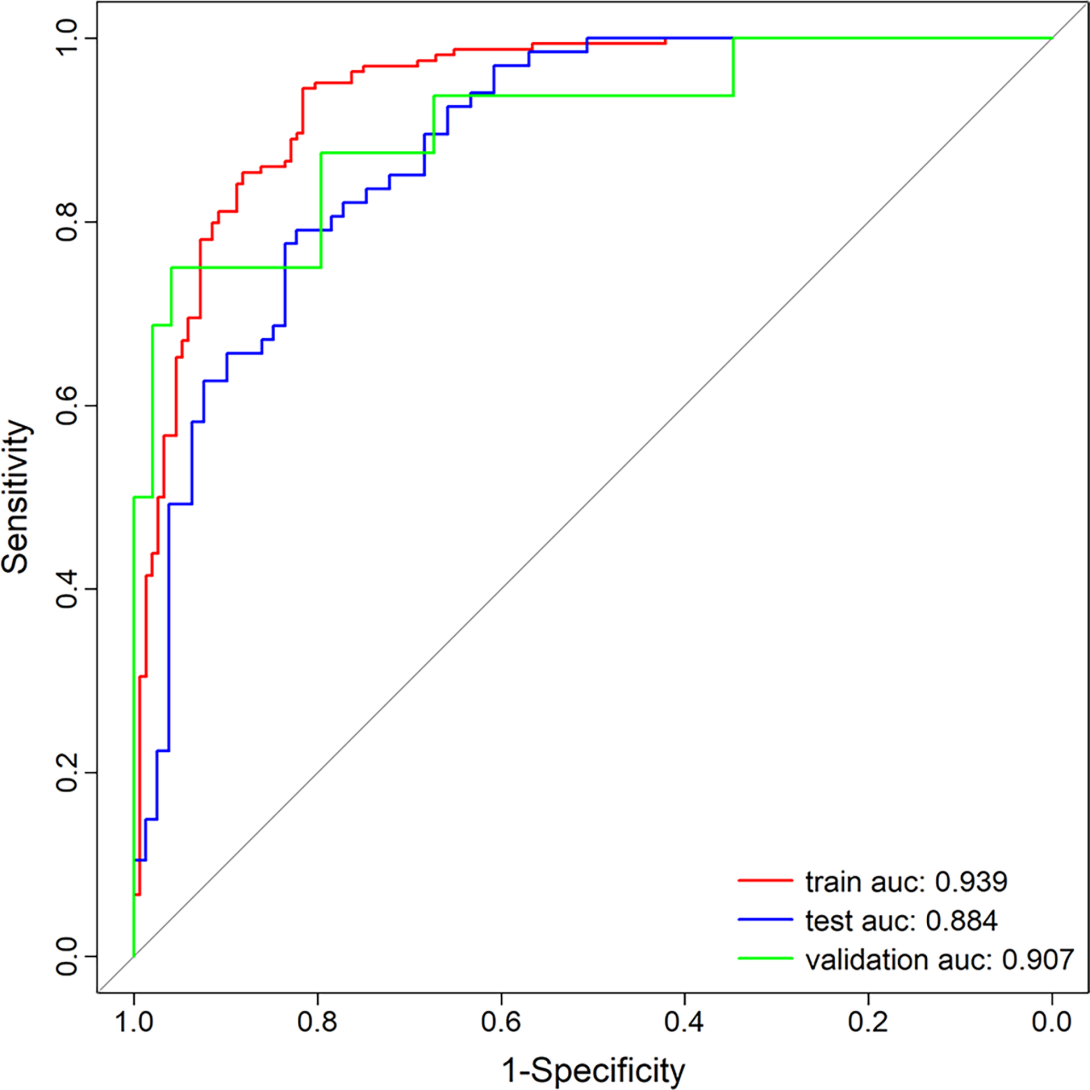
Receiver operating characteristic (ROC) curves of the radiomics nomogram.

The calibration curve of the nomogram demonstrated good agreement between prediction and observation in the training, test, and external validation groups; and the Hosmer–Lemeshow test showed a non-significant *p*-value of 0.248, 0.220, and 0.801, respectively (shown in **Figures 5A**–**C**). The AUC for the radiomics nomogram was 0.939 (95% CI, 0.913–0.965) for the training group, 0.884 (95% CI, 0.831–0.937) for the test group, and 0.907 (95% CI, 0.814–1) for the external validation group (shown in **Figure 6** and **Table 3**).

**Table 3:** Evaluation of radiomics nomogram.

	AUC	SEN	SPE	ACC
Training group	0.939 (0.878–0.941)	0.945	0.816	0.883
Test group	0.884 (0.831–0.937)	0.791	0.823	0.808
External validation group	0.907 (0.814–1)	0.75	0.959	0.908

AUC, area under the curve; SEN, sensitivity; SPE, specificity; ACC, accuracy.

**Figure 7:**
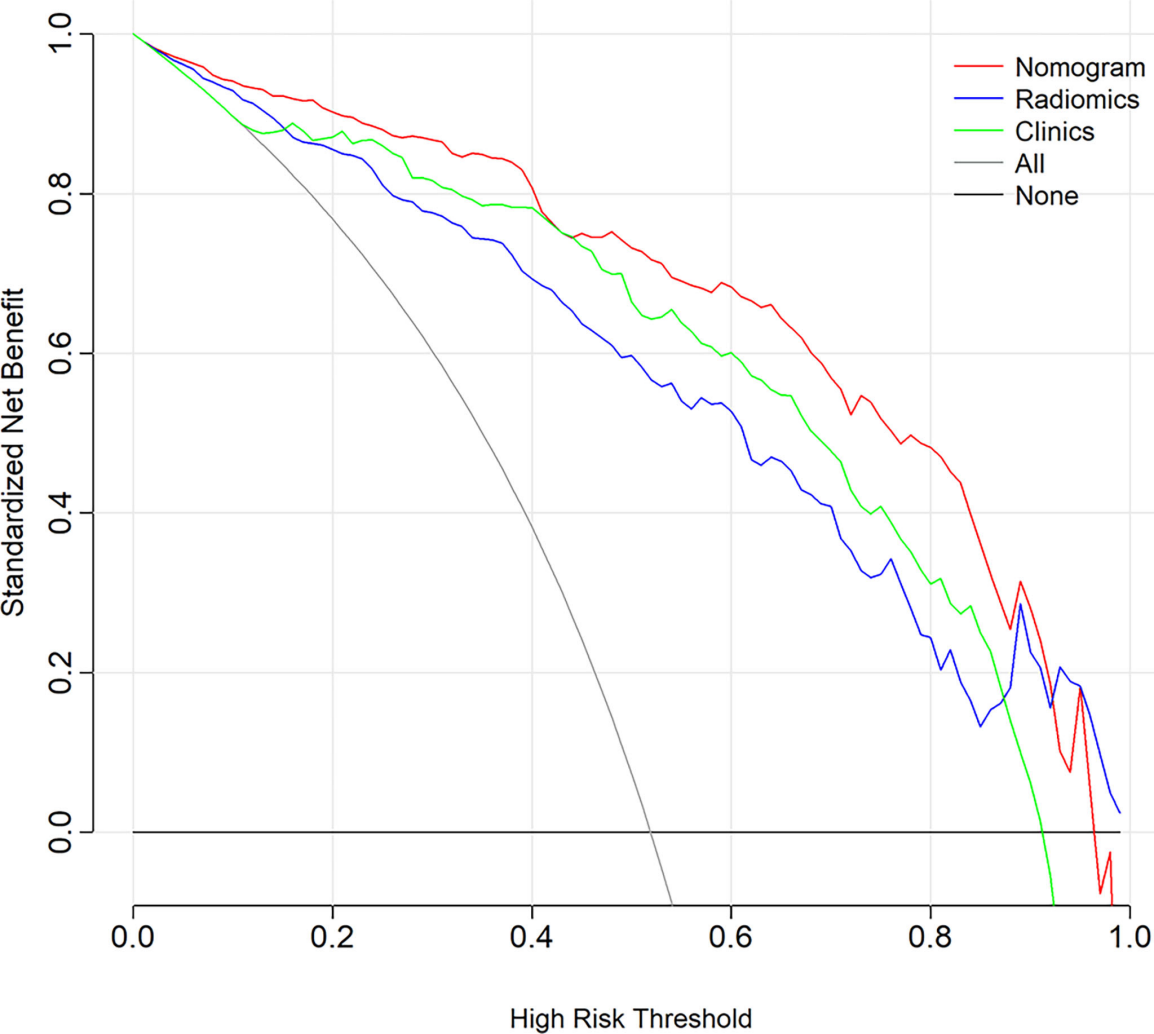
Decision curve analysis for the radiomics nomogram.

The DCA showed that if the threshold probability is higher than 0.05, the patients would obtain the greatest benefit using the radiomics nomogram than either the “treat all” strategy or the “treat none” strategy (**Figure 7**).

## Discussion

In the present study, we developed and validated a radiomics nomogram to diagnose CsPCa from non-CsPCa in PI-RADS 3 lesions. The radiomics nomogram was constructed by containing the Rad-score and PSAD. Compared with the radiomics signature, the radiomics nomogram acquired higher performance (AUC = 0.884 vs. 0.840 in the test group; AUC = 0.907 vs. 0.834 in the external validation group) in diagnosing CsPCa. Furthermore, the radiomics nomogram provided clinicians with an easy-to-use, quantifiable, and individualized tool to predict the risk rate of CsPCa in PI-RADS 3 lesions (**Figure 4**).

The class imbalance would adversely impact the performance of a classifier, leading to an unfair classification wherein all of the samples are classified as the majority class. To reduce the effect of imbalance, the SMOTE method has been used in the construction of radiomics signatures (27, 28). Feher et al. (24) have compared the performance of recursive feature elimination support vector machine (RFE-SVM) with or without oversampling, their study showed that RFE-SVM with SMOTE method has better performance for separating GS 6 versus GS ≥ 7, and GS 7 (3 + 4) versus GS 7 (4 + 3) cancers. Certainly, our results indicate that the performance of the radiomics signature with SMOTE method was improved in the test group (AUC = 0.840 vs. 0.730, *p* = 0.083) and external validation group (AUC = 0.834 vs. 0.718, *p* = 0.016).

Many studies have explored the radiomics analysis in oncology and extended to PCa identification and evaluation. Woznicki et al. (29) found that the radiomics model combined with clinical characters achieved high predictive performance (AUC = 0.844) for differentiation of CsPCa from clinically insignificant PCa (CiPCa) and superior to PI-RADS for CsPCa prediction. Our study has developed a nomogram to different CsPCa from no-CsPCa (including CiPCa and benign lesions), which showed higher performance (AUC = 0.907) than Woznicki et al. Zhang et al. (30) investigated an MRI-based radiomics nomogram for identifying CsPCa with an AUC of 0.95 (training group), 0.93 (internal validation group), and 0.84 (external validation group). In the present study, the AUC of the radiomics nomogram for predicting CsPCa was 0.939, 0.884, and 0.907 in the training group, test group, and validation group, respectively. The different results between Zhang et al. and our study may be illustrated by the differences in patient selection criteria and the different clinical risk factors included in the radiomics nomogram.

Although radiomics analysis has been proven to detect PCa and assess the aggressiveness of PCa, there is limited research evaluating radiomics in predicting CsPCa in PI-RADS 3 lesions. Hectors et al. (19) recently constructed a random forest classifier based on T2WI radiomics features to predict CsPCa within PI-RADS 3 lesion, and the AUC was 0.76 in the test set. Giambelluca et al. (18) evaluated the performance (AUC = 0.82) of texture analysis based on T2WI radiomics in diagnosing CsPCa in PI-RADS 3 lesions. Christopher et al. (20) showed a low ability (AUC = 0.68) of machine learning based on ADC radiomics features to diagnose CsPCa. Hou et al. (21) developed T2WI, DWI, and ADC radiomics machine learning models to predict CsPCa in PI-RADS 3 lesions with AUC of 0.89. The results of the above studies are quite different, maybe because the MRI sequence used in their studies is different. In the above studies, there were no validation data performed and even only Hectors et al. have conducted internal validation in their study. The main strengths of our study were that the inter- and extra-validation cohorts were included, and the radiomics nomogram achieved good prediction performance in both the test group (AUC = 0.884) and the external validation group (AUC = 0.907).

This study has several limitations. First, this is a retrospective study with a small study population. Falagario et al. (31) found that the accuracy of mpMRI in staging PCa was similar in different populations, indicating that our model may be valid also in other populations. Therefore, a larger sample size from multiple centers and different populations will be needed to validate the performance of the model in future studies. Second, ROIs were manually segmented by the radiologist. Although we have evaluated the repeatability of segmentation with resulting high ICC values, there might still exist subjective bias in the segmentation results, which could influence the stability and repeatability of the study findings. In future work, automatic segmentation should be used to address this problem. In addition, the peripheral zone and transition zone lesions were not separately analyzed given the small number of CsPCa in the study; evaluating radiomics features classified for each zone is warranted in future studies.

In conclusion, this study presented an mpMRI-based radiomics nomogram that incorporates both radiomics signature and PSAD for discriminating CsPCa from non-CiPCa among PI-RADS 3 lesions. The nomogram has great clinical application and provided a visual and individualized tool for the diagnosis of CsPCa in PI-RADS 3 lesions.

## Data Availability Statement

The original contributions presented in the study are included in the article/**Supplementary Material**. Further inquiries can be directed to the corresponding author.

## Ethics Statement

Written informed consent was not obtained from the individual(s) for the publication of any potentially identifiable images or data included in this article.

## Author Contributions

TL and PW conceived of the presented idea. LS, XL, and ML collected the data. TL, LS, and QL analyzed the data. TL drafted the manuscript. All authors reviewed the manuscript and PW made corrections to the manuscript. All authors contributed to the article and approved the submitted version.

## Funding

We acknowledge funding and support from the Natural Foundation of Shandong Province (ZR2018MH034).

## Conflict of Interest

The authors declare that the research was conducted in the absence of any commercial or financial relationships that could be construed as a potential conflict of interest.

## Publisher’s Note

All claims expressed in this article are solely those of the authors and do not necessarily represent those of their affiliated organizations, or those of the publisher, the editors and the reviewers. Any product that may be evaluated in this article, or claim that may be made by its manufacturer, is not guaranteed or endorsed by the publisher.

## Supplementary Material

Supplementary Table 1 MRI protocols for Institution 1 and Institution 2. TR, repetition time; TE, echo time; FA, flip angle; FOV, field of view.

Supplementary Table 2 Patients characteristics between training and test group. tPSA, total prostate-specific antigen; fPSA, free prostate-specific antigen; PSAD, prostate-specific antigen density.

Supplementary Figure 1 Coefficients of the selected features from LASSO regression. **(A)** Without SMOTE method; **(B)** With SMOTE method.
