# Supplementary material for: Development and Validation of a Radiomics Nomogram for Predicting Clinically Significant Prostate Cancer in PI-RADS 3 Lesions
Source: Front Oncol. 2022 Jan 26;11:825429. doi: 10.3389/fonc.2021.825429 (PMC8825569; doi:10.3389/fonc.2021.825429)
Supplement: Supplementary Table 1 — MRI protocols for Institution 1 and Institution 2. TR, repetition time; TE, echo time; FA, flip angle; FOV, field of view. [file DataSheet_1.docx]

**Supplementary Materials**

**Table S1.** MRI protocols for Institution 1 and Institution 2

|  | Institution 1  (GE Discovery MR750W) | | |  | Institution 2  (Siemens MAGNETOM Spectra) | | |
| --- | --- | --- | --- | --- | --- | --- | --- |
|  | T_2_WI | DWI | DCE |  | T_2_WI | DWI | DCE |
| TR (ms) | 5002 | 4994 | 4.32 |  | 4400 | 4200 | 3.22 |
| TE (ms) | 90.9 | 97.4 | 1.7 |  | 96 | 56 | 1.26 |
| FA (°) | 111 | 90 | 15 |  | 160 | 90 | 9 |
| Averages | 2 | 1 | 0.7 |  | 2 | 8 | 1 |
| FOV (mm^2^) | 280×280 | 224×224 | 304×304 |  | 240×240 | 262×300 | 292×360 |
| Matrix (px^2^) | 256×320 | 128×128 | 224×288 |  | 308×512 | 112×128 | 218×384 |
| Bandwidth (Hz) | 122.1 | 1953.1 | 325.1 |  | 200 | 2055 | 590 |
| Slice thickness (mm) | 4 | 4 | 5 |  | 3.5 | 5 | 2.5 |
| Slice gap (mm) | 5 | 5 | 2.5 |  | 4.2 | 5.5 | 0 |
| b-values (s·mm^-2^) |  | 0,50,800,1000 |  |  |  | 0,800,1000 |  |

TR, repetition time; TE, echo time; FA, flip angle; FOV, field of view.

**Table S2.** Patients characteristics between training and test group

|  | Training group  (n = 199) | Test group  (n = 107) | *P* |
| --- | --- | --- | --- |
| Age (years) | 69.76±7.87 | 70.90±7.97 | 0.233 |
| tPSA (ng/mL) | 13.30(7.44-25.92) | 13.63(7.21-27.01) | 0.752 |
| fPSA (ng/mL) | 1.61(1.01-2.86) | 1.74(1.06-2.94) | 0.761 |
| PSAD (ng/mL/cm^3^) | 0.20(0.10-0.52) | 0.24(0.13-0.64) | 0.455 |
| Gleason score (GS): |  |  | 0.274 |
| Benign | 130 | 60 |  |
| GS ≤ 6 | 28 | 13 |  |
| GS = 7 | 26 | 22 |  |
| GS = 8 | 11 | 10 |  |
| GS = 9 | 4 | 2 |  |


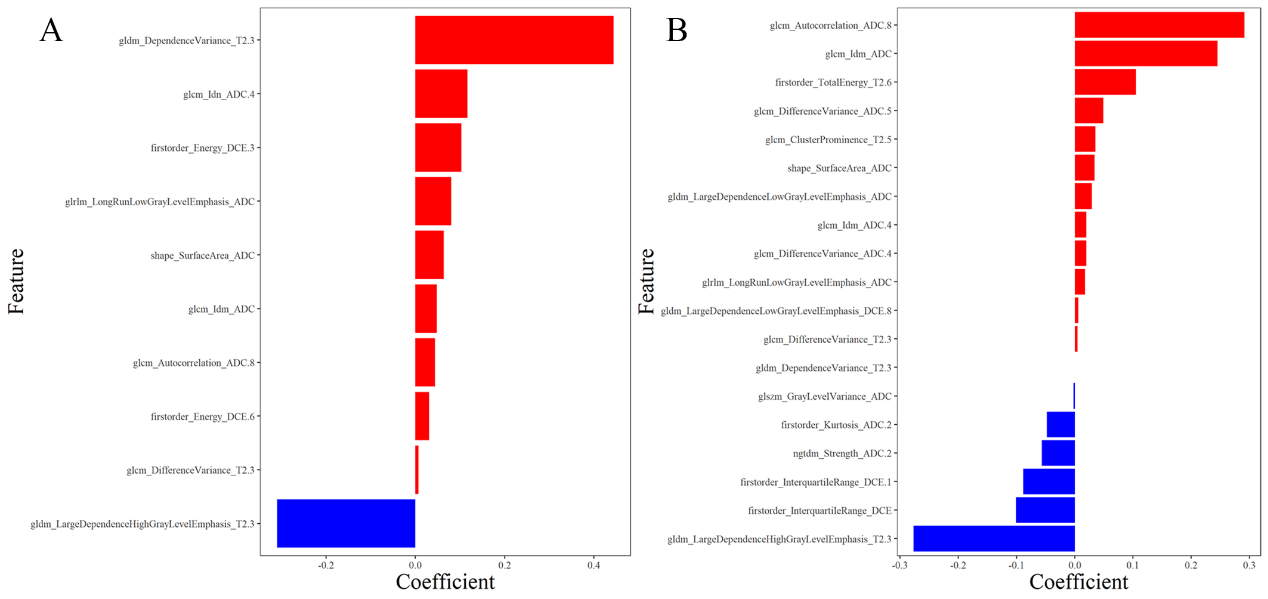


**Figure S1.** Coefficients of the selected features from LASSO regression. (A) Without SMOTE method; (B) With SMOTE method.
